# Reduction of Type IV Collagen by Upregulated *miR-29* in Normal Elderly Mouse and *klotho*-Deficient, Senescence-Model Mouse

**DOI:** 10.1371/journal.pone.0048974

**Published:** 2012-11-06

**Authors:** Masaki Takahashi, Akiko Eda, Tatsunobu Fukushima, Hirohiko Hohjoh

**Affiliations:** 1 National Institute of Neuroscience, NCNP, Kodaira, Tokyo, Japan; 2 Yokohama Research Laboratories, Mitsubishi Rayon Co., LTD., Tsurumi-ku, Yokohama, Japan; UMDNJ-New Jersey Medical School, United States of America

## Abstract

MicroRNA (miRNA), a small non-coding RNA that functions as a mediator in gene silencing, plays important roles in gene regulation in various vital functions and activities. Here we show that the *miR-29* members are upregulated in *klotho*-deficient [*klotho(−/−)*] mice, a senescence-model animal, and also in normal elderly ICR mice relative to wild-type littermates and young ICR mice. In addition, levels of type IV collagen, a major component of basement membranes and a putative target of *miR-29*, were lower in *klotho(−/−)* and elderly ICR mice than in wild-type littermates and young ICR mice. RNA degradation mediated by *miR-29* may participate in the suppression of type IV collagen, both *in vivo* and *in vitro*. Taken together, our current findings suggest that the *miR-29* upregulated in aging may be involved in the downregulation of type IV collagen, leading to a possible weakening of the basal membrane in senescent tissues, and *miR-29* may be a useful molecular marker of senescence.

## Introduction

Senescence is a set of biological changes that occurs in an individual with increasing age. A common change is the decline of physiology such as a decrease in hearing, visual activity, and memory skills; and behind such a physiological decline, there are presumably biochemical changes involved. In order to understand such complex senescent phenomena at a molecular level, finding biological molecules that show quantitative and qualitative variation with increasing age is vital; and such molecules may be useful as senescence makers.

MicroRNA (miRNA) is a 21∼23-nucleotide-long small non-coding RNA and functions as a mediator in gene silencing. Hundreds of miRNA genes have been found in animals and plants [see the microRNA database (miRBase): http://www.mirbase.org/index.shtml]. miRNAs play essential roles in gene regulation by inhibiting translation of messenger RNAs (mRNAs) that are partially complementary to the miRNAs, and by digestion of mRNAs that are nearly complementary to the miRNAs, or by RNA interference (RNAi), during cell proliferation, differentiation and development [1]–[5]. Expression profiles of miRNAs are quite useful in understanding complex gene regulation involving miRNAs and in characterizing miRNAs themselves. Many expression analyses have been performed in various tissues and cells, and revealed tissue- and stage-specific expression of miRNAs. In mammals, it has been found that a major small RNA class transition from retrotransposon-derived small interfering RNAs (siRNAs) and Piwi-interacting RNAs (piRNAs) to zygotically expressed miRNAs occurs during early development of pre-implantation embryos [6], and tissue- or organ-specific expression patterns of miRNAs are generated thereafter [4], [7]–[9].

Age-associated change in gene expression involving miRNAs is an important research field. We investigated the expression profile of miRNAs in mouse brain tissue by means of DNA chip and reverse-transcription quantitative polymerase chain reaction (RT-qPCR), and found that the alteration of the miRNA expression occurred during the course of mouse brain growth [10].

In this study we investigated miRNAs in *klotho*-deficient [*klotho(−/−)*] mice, which are a senescence model showing a number of phenotypes resembling human premature aging [11], and also miRNAs in 1 month and 14–18 month old outbred ICR mice studied as normal young and elderly mice, respectively. The data indicated that the *miR-29* members were markedly increased in *klotho(−/−)* and elderly ICR mice compared with wild-type littermates and young ICR mice.

## Materials and Methods

### Mice

*Klotho*-deficient male mice and ICR male mice were purchased from CLEA Japan, Inc (Tokyo, Japan). Mice were housed, fed and maintained in the laboratory animal facility according to the National Institute of Neuroscience animal care guidelines. This study was carried out in strict accordance with the recommendations in the Guide for the Care and Use of Laboratory Animals of the National Institutes of Neuroscience. The protocol was approved by the Committee on the Ethics of Animal Experiments of the National Institutes of Neuroscience (Permit Number: 2012003).

### DNA and RNA oligonucleotides

DNA oligonucleotides and small RNA duplexes used in this study were synthesized by and purchased from Sigma-Aldrich (St Louis, MO, USA).

### DNA chip analysis

*Genopal*®-MICM DNA chips (Mitsubishi Rayon, Tokyo, Japan) for detection of mouse miRNAs were used. DNA oligonucleotide probes for 200 miRNAs are installed on the DNA chip, and the list of the detectable miRNAs is available at the following address: http://www.mrc.co.jp/genome/pdf/micm07_list.pdf

Tissues were surgically removed from sacrificed mice and total RNAs were immediately isolated from the fresh tissues using TRIzol reagent (Invitrogen, Carlsbad, CA, USA) according to the manufacturer's instructions. Preparation of small-sized RNAs, fluorescent labeling, hybridization with DNA chips and detection of hybridization signals were carried out as described previously [10], [12], [13].

### Construction of reporter genes and hydrodynamic delivery method

To examine the knockdown potencies of endogenous *miR-29a* and *miR-29b in vivo*, we constructed reporter genes with the psiCheck2 vector (Promega): the *Renilla luciferase* gene carrying the complementary (target) sequence of *miR-29a* or *miR-29b* in the 3′ untranslated region was constructed as described previously [14]. The target sequences synthesized in the construction were as follows:

For the target of *miR-29a*;

Sense-strand; 5′- TCGAGTAACCGATTTCAGATGGTGCTATTACTAGT-3′
Antisense-strand; 5′- ACTAGTAATAGCACCATCTGAAATCGGTTAC-3′

For the target of *miR-29b*;

Sense-strand; 5′- TCGAGAACACTGATTTCAAATGGTGCTATTACTAGT-3′
Antisense-strand; 5′- ACTAGTAATAGCACCATTTGAAATCAGTGTTC-3′

The reporter plasmid and the pSV-β-Galactosidase control vector (Promega) as a control were administered to young (5-week-old) and elderly (8∼18-month-old) ICR mice by a hydrodynamic delivery method. Briefly, 100 µg of the reporter plasmid and 40 µg of the pSV-β-Galactosidase control vector were mixed in 2 ml of sterile saline solution. ICR mice were anesthetized with somnopentyl (50 mg/kg b.w.), and 2 ml of the plasmid mixture was systemically administered via the lateral tail vein within 5 seconds. Three days after administration, the heart, lung, liver and kidney were excised from the mice, and the tissue extracts were prepared. The expression levels of the *luciferase* and *β-galactosidase* reporter genes were measured using a Dual-Luciferase Reporter Assay System (Promega) and a Beta-Glo Assay System (Promega), respectively, by the Fusion Universal Microplate Analyzer (PerkinElmer, Waltham, MA, USA).

### Systemic administration of the synthetic *miR-29b* duplex

The synthetic *miR-29b* duplex and siControl (non-silencing siRNA; QIAGEN, Venlo, Netherlands) were each prepared with atelocollagen (AteloGene Systemic Use; KOKEN, Tokyo, Japan) according to the manufacturer's instructions. The resultant RNA/atelocollagen complexes (1.0 mg/kg b.w.) were systemically administered to normal male ICR mice (5-week-old; n = 5/group) via the lateral tail vein. Four systemic administrations were carried out (Day 1, 4, 8, and 12), and two days after the last administration (Day 14) the treated mice were examined. The sequences of the synthetic *miR-29b* duplexes were as follows:

Sense- strand; 5′- UAGCACCAUUUGAAAUCAGUGUU-3′
Antisense-strand; 5′- CACUGAUUUCAAAUGGUGUUAUU-3′

### Cell culture, transfection of *miR-29* mimics and cell viability assay

Neuro2a (N2a), a mouse neuroblastoma cell line originally established by RJ Kleber and FH Ruddle (1969) [15], was used in this study. Human embryonic kidney 293 (HEK293) cells were obtained from the Health Science Research Resources Bank. The cells were grown in Dulbecco's modified Eagle's medium (Wako, Osaka, Japan) supplemented with 10% fetal bovine serum (Invitrogen), 100 U/ml penicillin and 100 µg/ml streptomycin (Wako) at 37°C in 5% CO_2_-humidified chamber as described previously [13], [16], [17]. The day before transfection, cells were trypsinized, diluted with culture medium without antibiotics, and seeded into 96-well culture plates at a cell density 5×10^4^ cells/cm^2^. To each well, 20 nM (final concentration) of each MISSION microRNA mimic of *has-miR-29a*, *has-miR-29b*, or negative control miRNA (Sigma-Aldrich) was transfected into the cells using Lipofectamine2000 transfection reagent (Invitrogen) according to the manufacturer's instructions. Four hours after transfection, the culture medium was replaced with fresh medium containing antibiotics. After the 3-day incubation, total RNA isolation as described above or cell viability assay was carried out. Cell viability was examined with the CellTiter 96 AQueous One Solution Cell Proliferation Assay (Promega, Fitchburg, WI, USA) according to the manufacturer's instructions.

For assessment of the knockdown potency of *miR-29a* or *miR-29b*, the constructed reporter plasmids (see above) together with or without synthetic *miR-29* duplexes were transfected into N2a cells, and 24 hour after transfection the dual luciferase assay was carried out as described previously [14].

### Reverse transcription (RT)-(real time)-polymerase chain reaction (PCR)

Total RNAs extracted by TRIzol reagent (Invitrogen) were treated with Turbo DNase I (Applied Biosystems, Carlsbad, CA, USA) according to the manufacturer's instructions and subjected to RT-(real time)-PCR. Real time PCR (quantitative PCR; qPCR) was performed using the AB 7300 Real Time PCR System (Applied Biosystems) with a TaqMan Universal PCR Master Mix together with TaqMan® MicroRNA Assays (Applied Biosystems) according to the manufacturer's instructions.

The TaqMan® MicroRNA Assays used were as follows (ABI Assay IDs are indicated in parentheses):

*hsa-miR-29a* (2112), *hsa-miR-29b* (413) and *snoRNA202* (1232).

For examination of protein-coding genes, total RNAs were subjected to cDNA synthesis using Oligo(dT)_15_ primers (Promega) and a SuperScript III reverse transcriptase (Invitrogen) according to the manufacturer's instructions. The cDNAs were examined by qPCR using the AB 7300 Real Time PCR System (Applied Biosystems) with SYBR Green PCR Master Mix (Applied Biosystems) and TaqMan Universal PCR Master Mix together with Perfect Real Time Primers (TAKARA BIO, Otsu, Shiga, Japan) and TaqMan® Gene Expression Assays (Applied Biosystems), respectively, according to the manufacturer's instructions.

The Perfect Real Time Primers used were as follows (TAKARA BIO primer-set IDs are indicated in parentheses):

*Col4a1* (MA118711), *Col4a2* (MA112149), *Col4a3* (MA124802), *Col4a4* (MA077950), *Col4a5* (MA113961), *Col4a6* (MA087283), *Cabin1* (MA069171), *Dnmt1* (MA060016), *Dicer1* (MA043537), *Lamc1* (MA059108), *Gapdh*, (MA050371), *COL4A1* (HA156982), *COL4A2* (HA174126), *CABIN1* (HA183487), *DNMT1* (HA002770), *DICER1* (HA174786), *LAMC1* (HA083467) and *GAPDH* (HA067812).

TaqMan® Gene Expression Assays used were as follows (ABI Assay IDs are indicated in parentheses):

*klotho* (Mm00502002_m1) and *Gapdh* (Mm99999915_g1).

### Western blotting

Equal amounts of protein was separated by SDS-PAGE and electrophoretically blotted onto PVDF membranes (Millipore, Billerica, MA, USA). Membranes were blocked in blocking solution (4% bovine serum albumin and 0.05% Tween-20 in TBS) and incubated with rabbit polyclonal anti-collagen IV (mouse) antibody (250485; ABBIOTEC, San Diego, CA, USA) or mouse monoclonal anti-α-tubulin antibody (Sigma-Aldrich), followed by washing in TBS containing 0.05% Tween-20, and then incubated with horseradish peroxidase-conjugated anti-rabbit or anti-mouse anti-goat IgG. Antigen-antibody complexes were visualized using a chemiluminescent reagent (Millipore).

### Blood tests

Blood samples drawn from mice were subjected to biochemical tests. To examine renal function, plasma creatinine levels were measured with a creatinine assay kit (BioVision Inc., Milpitas, CA, USA). The plasma levels of hexanoyl-lysine adduct (HEL) and 8-hydroxy-2′-deoxyguanosine (8OH-dG) as stress markers were measured by HEL ELISA Kit [Japan Institute for the Control of Aging (JaICA), NIKKEN SEIL Co., Ltd., Shizuoka, Japan] and High Sensitive 8-OHdG Check ELISA Kit (JaICA), respectively.

### Statistical analysis

Differences in the levels of expression of miRNAs were analyzed by Student's *t*-test (two-tailed).

### Accession numbers

The Gene Expression Omnibus (GEO) accession numbers for the miRNA expression data used in this study are GSE34531 (miRNAs in *Klotho(−/−)* and wild-type mouse tissues) and GSE34532 (miRNAs in young and elderly ICR mouse tissues).

## Results

### miRNA expression profiles of *klotho*-deficient mice

The *klotho* gene encodes a single-pass transmembrane protein involved in the maintenance of homeostasis, and *klotho*-deficient [*klotho*(−/−)] mice are known to be an model of aging that shows various senescence-like phenotypes resembling human premature aging such as a short lifespan, decreased bone mineral density and atrophy of lymphopoietic and reproductive organs [11]. We investigated miRNAs in *klotho*(−/−) mice and wild-type littermates by means of DNA chip. Duplicated DNA chip analyses with different individual mice exhibited reproducible results except for the data of the *klotho*(−/−) spleen (Fig. S1); the discrepancy for the spleen miRNAs may be due to individual differences.

Fold changes (FC) in the expression levels of miRNAs between *klotho*(−/−) and wild-type mice were calculated, and the miRNAs that showed more than 1.5 FC values or less than 0.5 FC values are listed (Table 1 and Table S1): the former (>1.5 FC) represents the miRNA that were markedly increased in *klotho*(−/−) mice versus wild-type mice, and the latter (<0.5 FC) represents the miRNA markedly decreased in *klotho*(−/−) mice. From the FC analysis, the *miR-29* members (*miR-29a, -29b and -29c*) appear to be commonly upregulated in *klotho*(−/−) mice (Table 1).

**Table 1:** Difference in the expression of miRNAs between *klotho*-deficient and wild-type mice.

		Exp.1			Exp.2		
Tissue	miRNA	*Kl*	Wt	FC	*Kl*	Wt	FC
Lung	mmu-miR-29b	791	332	2.38	567	150	3.79
	mmu-miR-29c	502	256	1.96	449	133	3.37
	mmu-miR-409	42	22	1.88	41	26	1.55
	mmu-miR-296	124	67	1.86	122	45	2.71
	mmu-miR-29a	978	575	1.70	899	266	3.38
	mmu-miR-101a	228	147	1.55	214	88	2.43
Liver	mmu-miR-29b	171	22	7.70	122	39	3.13
	mmu-miR-29a	216	47	4.60	154	67	2.30
	mmu-miR-29c	106	25	4.30	90	36	2.48
	mmu-miR-145	46	14	3.35	31	16	1.89
	mmu-miR-101a	102	32	3.19	77	51	1.52
	mmu-miR-27a	104	34	3.09	75	49	1.51
	mmu-miR-24	120	42	2.86	89	57	1.57
	mmu-miR-126-3p	97	36	2.71	72	48	1.50
	mmu-miR-26a	219	84	2.62	198	121	1.63
	mmu-miR-101b	131	55	2.40	116	75	1.56
	mmu-miR-30b	44	19	2.33	37	24	1.56
Kidney	mmu-miR-29a	437	158	2.76	361	192	1.88
	mmu-miR-29b	358	143	2.50	293	171	1.72
	mmu-miR-34a	99	41	2.41	105	49	2.16
	mmu-miR-31	17	7	2.34	15	10	1.53
	mmu-let-7b	1099	471	2.33	487	315	1.55
	mmu-miR-199a	65	29	2.23	51	33	1.54
	mmu-miR-92	54	25	2.20	34	19	1.83
	mmu-miR-125a	161	74	2.19	105	61	1.73
	mmu-miR-21	418	193	2.17	323	212	1.53
	mmu-miR-296	120	56	2.16	55	36	1.51
	mmu-miR-125b	212	101	2.10	152	94	1.61
	mmu-let-7c	1563	755	2.07	806	515	1.57
Heart	mmu-miR-298	64	4	15.66	27	5	5.88
	mmu-miR-291a-3p	35	2	15.50	18	2	7.49
	mmu-miR-370	30	4	7.05	13	6	2.02
	mmu-miR-328	39	10	4.03	13	8	1.63
	mmu-miR-150	39	16	2.46	25	15	1.64
	mmu-miR-194	18	8	2.28	14	7	1.86
	mmu-miR-29b	320	143	2.24	240	136	1.77
	mmu-miR-92	47	31	1.52	28	17	1.64
	mmu-miR-146	12	22	0.56	8	14	0.57

Hybridization signal intensities obtained from DNA chip analysis [duplicated experiments (Exp.) 1 and 2] are indicated.

Fold changes (FC) in the expression of miRNAs between *klotho* (*Kl*) and wild-type (Wt) mice are calculated.

### Upregulation of *miR-29* in elderly ICR mice

**Figure 1:**
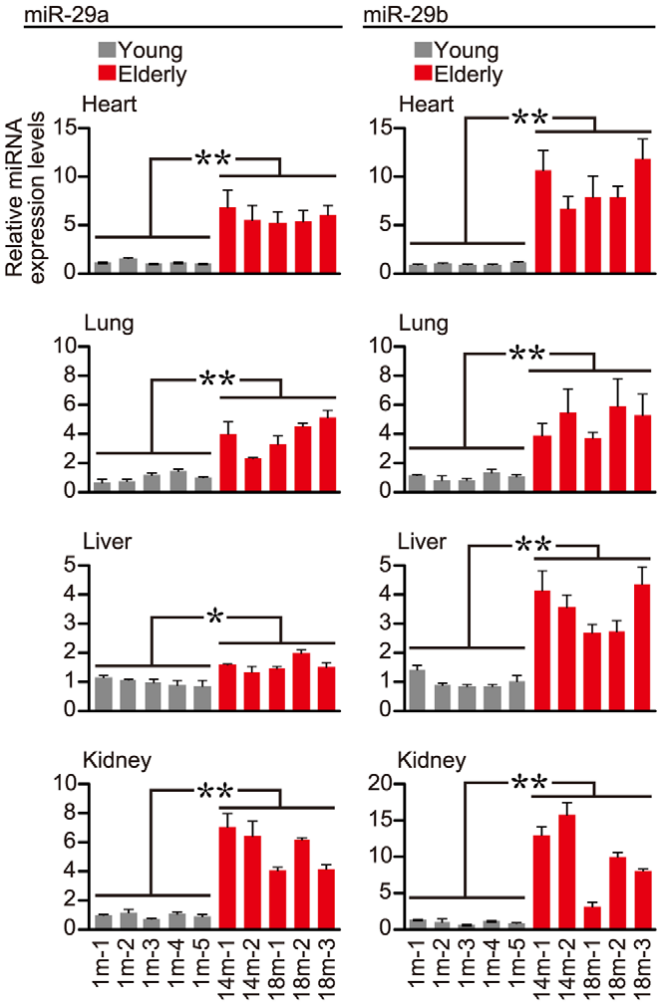
*miR-29a* and *miR-29b* expression in young and elderly ICR mice. Total RNAs were extracted from indicated tissues of young (1-month-old: 1 m) and elderly (14- or 18-month-old: 14 m or 18 m) ICR mice, and subjected to RT-qPCR analysis. The *miR-29a*, *miR-29b* and *snoRNA202* (as a control) expressions were examined and analyzed by the delta-delta Ct method. Five individual mice in each group were investigated to account for individual variability, and three measurements by qPCR per sample were carried out. Error bars represent standard deviations. The normalized *miR-29a* and *miR-29b* expression levels in the young (gray bars) and elderly (red bars) groups in each tissue were further examined by Student's *t*-test, and significant differences between the two groups in all the tissues examined were detected (* *P*<0.05, **P<0.01).

To see if normal aged-mice, like *klotho*(−/−) mice, have a similar increase in the expression of *miR-29*, we measured *miR-29a* and *miR-29b* in outbred ICR mice at age 14 or 18 months (14 m, 18 m; elderly mice) and 1 month (1 m; young mice) by means of RT-qPCR; note that five different individual ICR mice in each group were examined to account for individual differences. The results of the qPCR analysis consistently indicated that the levels of either *miR-29a* or *miR-29b* were significantly higher in elderly mouse tissues than in young tissues (Fig. 1).

**Figure 2:**
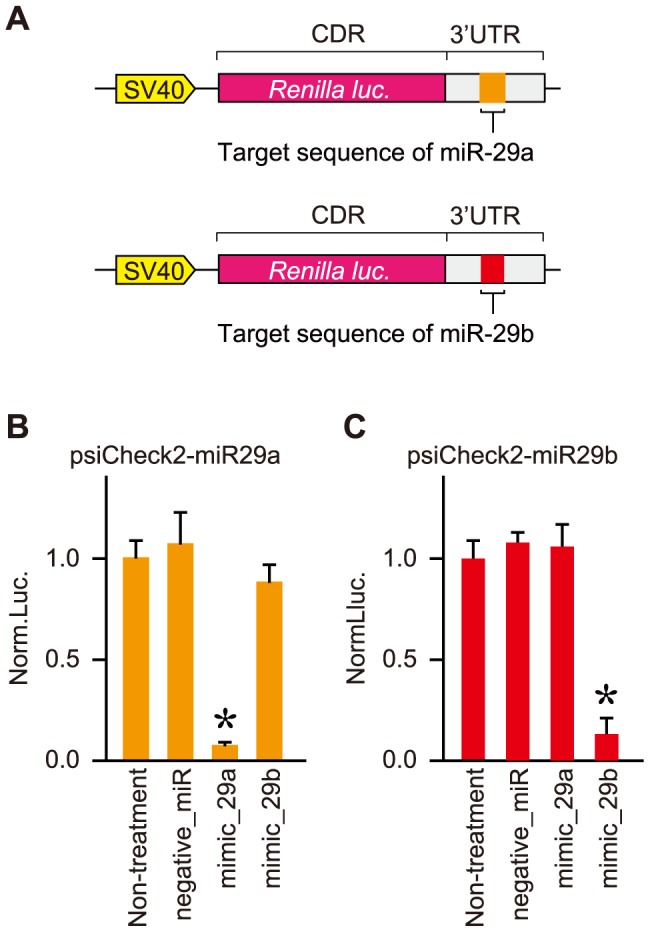
Assessment of knockdown potency using reporter genes. (**A**) Schematic drawing of constructed reporter genes. The psiCheck2-backbone reporter plasmid encoding the *Renilla luciferase* gene carrying the target sequence of *miR-29a* (psiCheck2-miR29a) or *miR-29b* (psiCheck2-miR29b) was constructed. The SV40 promoter, *Renilla luciferase* coding region (CDR) and 3′ untranslated region (3′UTR) of the reporter genes are indicated. The target sequences of *miR-29a* and *miR-29b* are indicated in the 3′UTRs. (**B, C**) Suppression of reporter genes by synthetic *miR-29* mimics. The reporter plasmids indicated were transfected together with synthetic miRNA mimics of *miR-29a* (mimic_29a), *miR-29b* (mimic_29b) or a negative control miRNA (negative_miR) into N2a cells. 24 hr after transfection, dual-luciferase assay was carried out. The target (*Renilla*) luciferase activity was normalized to control (*Photinus*) luciferase activity and further normalized to the data obtained from N2a cells transfected with only the reporter plasmid (non-treatment). Data are averages of four independent experiments. Error bars represent standard deviations. The normalized data were analyzed by one-way analysis of variance (ANOVA), followed by Dunnett's test. Significant differences are indicated by * (*P*<0.05).

**Figure 3:**
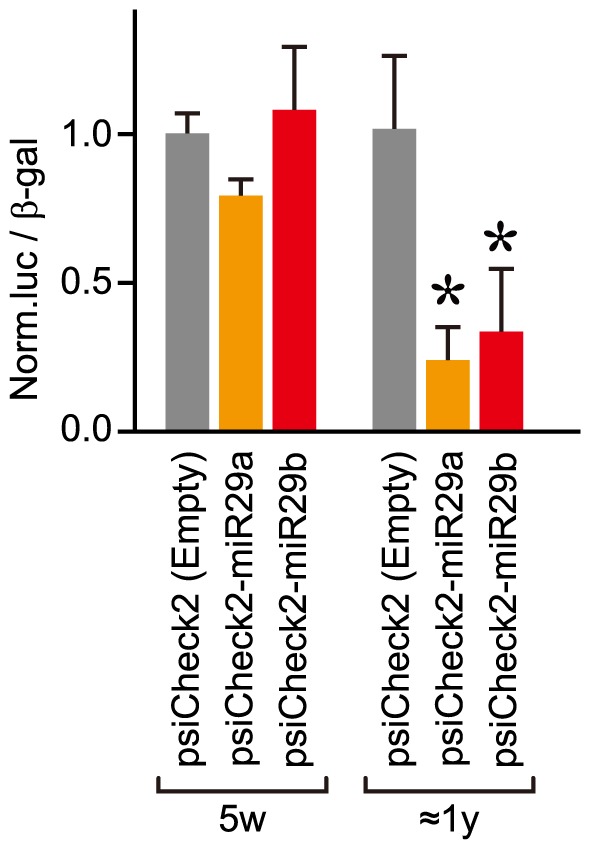
*In vivo* knockdown potency mediated by endogenous *miR-29a* or *miR-29b*. The constructed reporter plasmid carrying the *miR-29a* or *miR-29b* target sequence (Fig. 2A) and the *β-galactosidase* expression plasmid as a control were systemically administrated to young (5-week-old: 5w) and elderly (8∼18-month-old: ≈1 y) ICR mice by a hydrodynamic delivery method. Three days after administration, kidney cell extracts were prepared from the treated mice, and then the luciferase and β-galactosidase activities were examined. The expression levels of luciferase were normalized to those of β-galactosidase, and further normalized to the data obtained with a control empty vector (Empty). Data are averages of three different individual's data. Error bars represent standard deviations. The normalized data in the young (5w) and elderly (≈1 y) groups were analyzed by Student's *t*-test (one-tailed), and significant differences from the data of each empty vector are indicated by * (*P*<0.05).

We further investigated whether the upregulated *miR-29* members in elderly mice enhanced their mediated knockdown activities. To address this, the *luciferase* reporter genes carrying the complementary (target) sequences of *miR-29a* and *miR-29b*, respectively, in their 3′ untranslated regions (3′UTRs) were constructed (Fig. 2), and administered to elderly and young ICR mice by a hydrodynamic delivery method. Three days after administration, the expression of the reporter gene was examined. Neither heart nor lung had expression of the *luciferase* reporter genes, whereas liver and kidney exhibited expression; however, the expression data in liver, particularly the levels of the *luciferase* carrying no target sequence (control empty vector), were poorly-reproducible, so that they were not able to be further evaluated (Fig. S2). The expression data for kidney were further normalized to the data obtained with the control empty vector. The normalized expression levels of the reporter gene carrying either the *miR-29a* or *miR-29b* target sequence was markedly decreased in elderly kidney than in young kidney (Fig. 3). Therefore, *miR-29* upregulation in elderly kidney may result in the enhancement of gene silencing mediated by it.

### *Klotho* expression in elderly ICR mice

**Figure 4:**
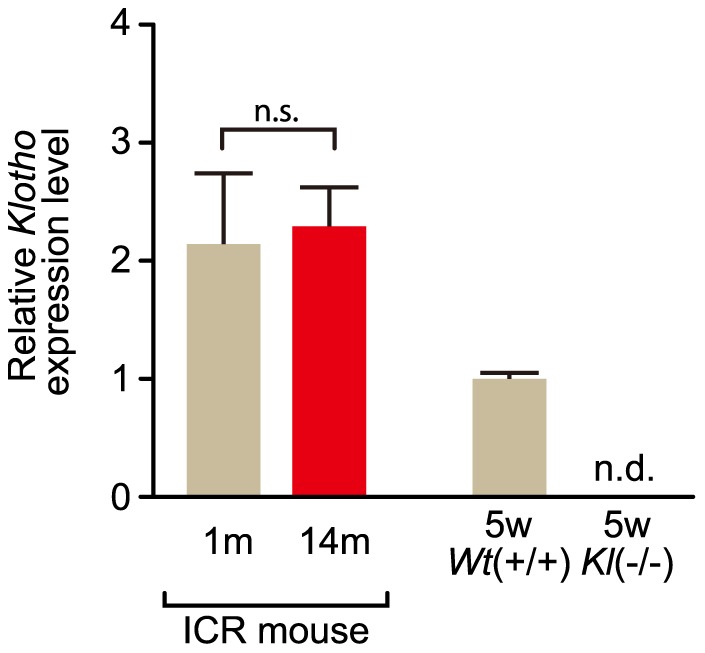
*Klotho* expression in young and elderly mice. Because *klotho* is predominantly expressed in kidney [11], *klotho* expression was examined in this tissue. Total RNAs were extracted from kidney of young (1-month-old: 1 m) and elderly (14-month-old: 14 m) ICR mice and also of 5-week-old (5w) *klotho*-deficient [*Kl(−/−)*] and wild-type littermate [*Wt(+/+)*] mice, and examined by RT-qPCR for *klotho* and *Gapdh*. Three measurements by qPCR per each sample were carried out. The expression levels of *klotho* and *Gapdh* (as a control) were analyzed by the delta-delta Ct method. Error bars represent standard deviations. n.s., no statistical significance; n.d., no detection.

The upregulation of *miR-29* members was seen in elderly ICR mice as well as *klotho*(−/−) mice lacking *klotho* expression; this raised the question of whether *klotho* might influence the expression of the *miR-29* members, although *klotho* is predominantly expressed in kidney, and hardly expressed in other tissues including heart, lung and liver. We investigated *klotho* expression levels in elderly and young ICR mice by RT-qPCR. The results indicate that there was no significant difference in the expression of *klotho* between elderly and young ICR mice (Fig. 4). Therefore, *miR-29* expression is probably unaffected by *klotho* expression levels.

### Target genes of *miR-29*

Target genes controlled by the *miR-29* upregulated in elderly mice may be also involved in natural aging. We searched for candidate gene targets of *miR-29* using the TargetScan program (http://www.targetscan.org/vert_40/), and selected the *type IV alpha collagen* gene family as a potential target. The *type IV collagen* gene family is composed of the *Col4α1*, *Col4α2*, *Col4α3*, *Col4α4*, *Col4α5* and *Col4α6* genes, encoding the α1, α2, α3, α4, α5 and α6 chains of type IV collagen, respectively; and the genes carry putative binding sequences of the *miR-29* members in their 3′UTRs. Additionally, previous studies with cultured mammalian cells suggest a significant association between *miR-29* members and the *type I* or *type IV collagen* genes [18]–[20].

**Figure 5:**
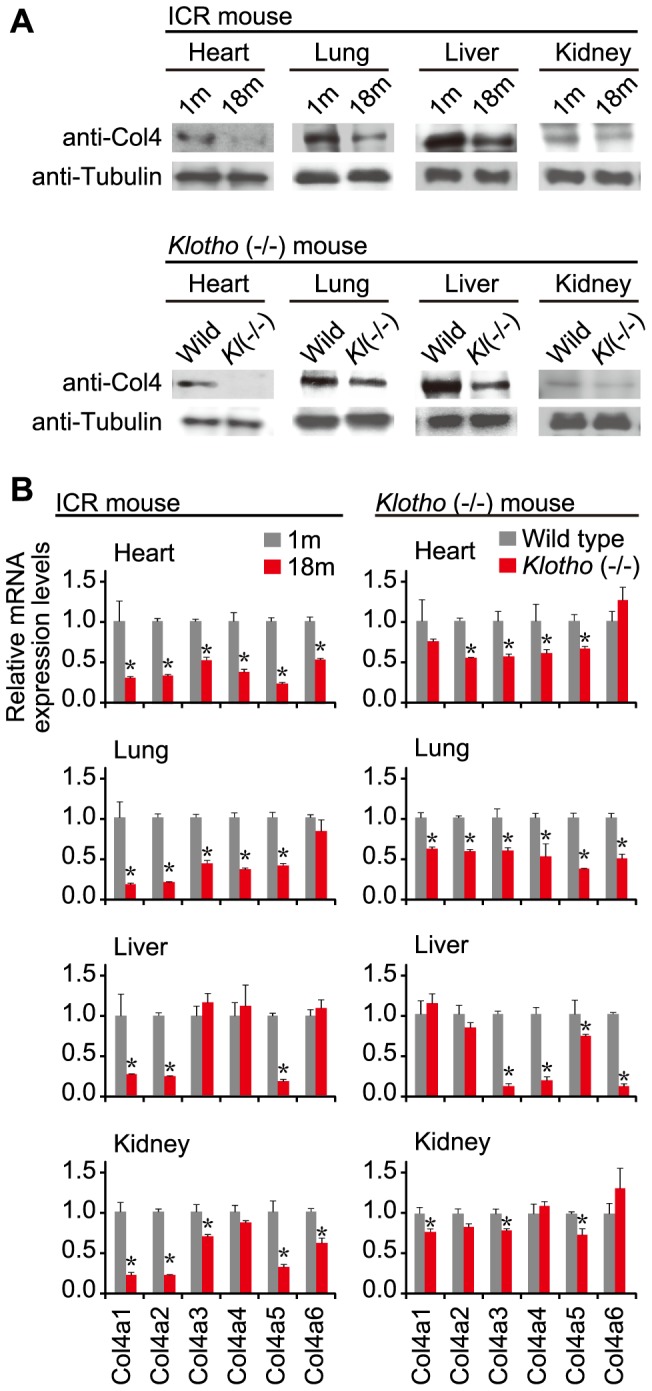
Expression profile of type IV collagen. (**A**) Western blot analysis of type IV collagen. Protein samples prepared from indicated tissues of young (1-month-old: 1 m) and elderly (18-month-old: 18 m) ICR mice and also of *klotho*(−/−) [Kl(−/−)] and wild-type littermate mice were examined by Western blotting with anti-collagen IV antibody (anti-Col4) and anti-α-tubulin antibody (anti-Tubulin) as an internal loading control. (**B**) Expression of *Col4α1–α6*. The expression levels of *Col4α1*–*α6* belonging to the *type IV collagen* gene family, and *Gapdh* as a control in indicated tissues of young (1 m) and elderly (18 m) ICR mice, and in *klotho*(−/−) and wild-type littermate mice were examined by RT-qPCR followed by analyses with the delta Ct method. The levels of *Col4α1–α6* expression were normalized to those of *Gapdh*, and further normalized to the data of young ICR mice or wild-type littermates, each of which was set as 1. Error bars represent standard deviations. The normalized expression data were analyzed by Student's *t*-test (two-tailed). Significant differences between young (1 m) and elderly (18 m) ICR mice and between *klotho*(−/−) and wild-type littermate mice are indicated by * (*P*<0.05).

Western blot analyses of type IV collagen indicated that the levels of type IV collagen in heart, lung, liver and kidney of elderly ICR mice was less than that of young mouse tissues (Fig. 5A; upper panel). Interestingly, similar results were also observed between *klotho*(−/−) and wild-type littermate mice (Fig. 5A; lower panel).

Next we examined the *Col4α1–α6* transcripts in the tissues by means of RT-qPCR. The levels of most of the transcripts in elderly ICR and *klotho*(−/−) tissues were less than those in young and wild-type littermate tissues, although there were a few exceptions (Fig. 5B). Based on the data, levels of type IV collagen appear to correlate with levels of *Col4α1–α6* transcripts.

### *In vitro* gene silencing mediated by synthetic *miR-29*

**Figure 6:**
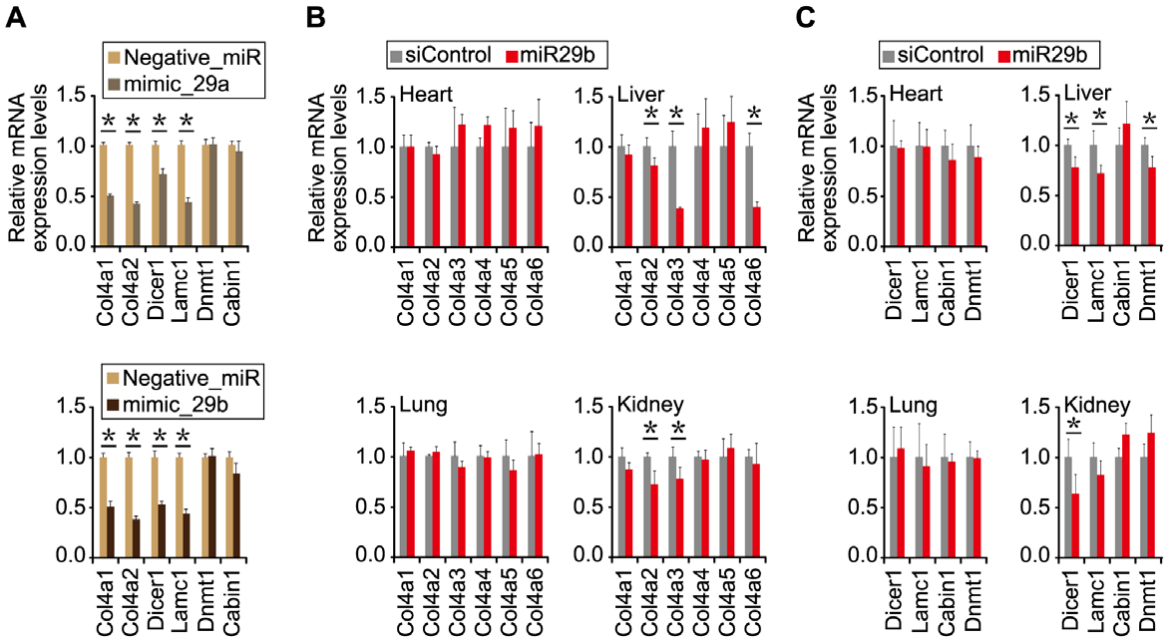
Gene expression profiles in *miR-29*-treated N2a cells and young ICR mice. (**A**) Expression profiles in *miR-29*-treated N2a cells. MISSION microRNA Mimic (Sigma-Aldrich) *has-miR-29a* (mimic_29a), *has-miR-29b* (mimic_29b) or negative control miRNA (negative_miR) was transfected into N2a cells. Three days after transfection, total RNAs were extracted and examined by RT-qPCR followed by analysis using the delta-delta Ct method using the expression levels of *Gapdh* as a control. The data were further normalized to the data obtained with the negative_miR. Error bars represent standard deviations. The genes examined are indicated: *Col4α1*, *Col4α2*, *Dicer1*, *Dnmt1*, *Lamc1* and *Cabin1*. The *Col4α3–α6* genes were examined as well, but their expression was not detected in the cells. (**B**, **C**) Gene expression profiles in *miR-29b*-treated ICR mice. Normal ICR mice (5-week-old) were subjected to four systemic administrations (Day 1, 4, 8, and 12) of *miR-29b* duplex or siControl (as a negative control). Two days after the last administration, the treated mice were examined. RT-qPCR analyses were carried out as in A. The examined tissues and genes are indicated: (**B**) *type IV collagen* gene family and (**C**) other genes. Statistically significant decrease in the expression of genes in the *miR-29b*-treated mice is indicated by * (*p*<0.05).

To verify whether the gene silencing mediated by *miR-29* could influence the regulation of *type IV collagen* gene expression, we introduced synthetic *miR-29a* (*miR-29a* mimic) or *miR-29b* (*miR-29b* mimic) into mouse Neuro2a (N2a) cells and examined their effects on gene silencing against endogenous *type IV collagen* and other genes (Fig. 6A). RT-qPCR analyses indicated that: (i) levels of *Col4α1* and *Col4α2* were significantly reduced by introduction of either *miR-29a* mimic or *miR-29b* mimic relative to the negative control, (ii) levels of *calcineurin binding protein 1* (*Cabin1*) and *DNA methyltransferase 1* (*Dnmt1*) genes carrying no putative binding sequence of *miR-29* remained almost unchanged, and (iii) levels of *Dicer1* and *laminin, gamma 1* (*Lamc1*) genes carrying a putative binding site for *miR-29* decreased in the presence of the *miR-29* mimic. Similar results were also obtained when human embryonic kidney 293 (HEK293) cells were used instead of N2a cells (Fig. S3). Therefore, these findings indicate that the gene silencing mediated by *miR-29* suppressed *type IV collagen*, *Dicer1* or *Lamc1*, each of which carries a putative binding site(s) of *miR-29*, and that RNA degradation may partially contribute to the gene silencing.

### *In vivo* gene silencing mediated by synthetic *miR-29b*

To further confirm the effects of *miR-29* on the expression of its target genes *in vivo*, we administered a synthetic *miR-29b* duplex or siControl duplex to 5-week-old (young) ICR mice. After four systemic administrations of the individual small RNA duplexes, the treated ICR mice were examined. RT-qPCR analyses indicated a significant decrease in the expression of some of the *Col4α* genes in liver and kidney of the *miR-29b*-treated mice compared with the siControl-treated mice (Fig. 6B). In contrast, no significant difference in the expression was seen in heart and lung between the mice (Fig. 6B); this may be due to the failure of delivery of the small RNA duplexes into these tissues as in the case of using the reporter plasmids described above. A similar decreasing tendency of the expression of *Dicer1* and *Lamc1* in liver and kidney of the *miR-29b*-treated ICR mice was also detected (Fig. 6C). Together with the results of Figs. 1 and 3, the findings suggest that the introduced synthetic *miR-29b* can enhance *in vivo* gene silencing against its target genes such as *Col4α*, *Dnmt1* and *Cabin1* even in young (5-week-old) ICR mice in which the endogenous *miR-29* is expressed at a low level. Accordingly, it is conceivable that the *miR-29* members spontaneously upregulated in elderly mice may have gradually contributed to such gene silencing (regulation) of target genes.

### Plasma biochemical analyses

**Figure 7:**
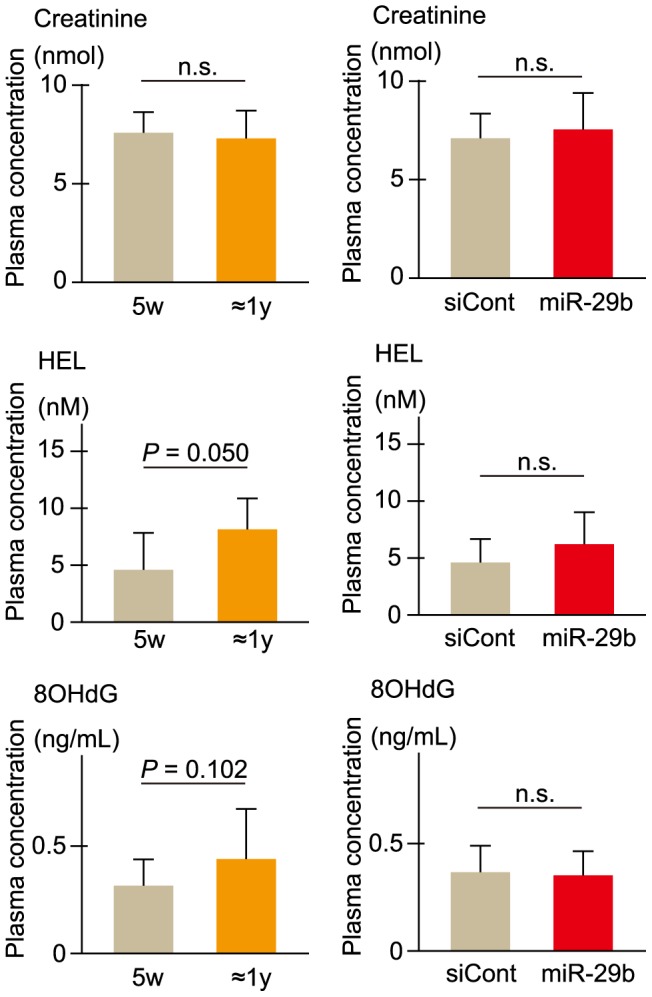
Plasma creatinine, HEL, and 8OHdG levels in elderly and young mice and *miRNA*-treated mice. Plasma samples were prepared from young (5-week-old; 5w) and elderly (8∼18-month-old: ≈1 y) ICR mice and also from miR-29b- and siControl (siCont)-treated ICR mice (5-week-old), and then subjected to plasma biochemical tests. The level of creatinine was measured as an index of renal function, and HEL and 8OHdG were each examined as a stress marker. Data are averages of three different individual's measurements. Error bars represent standard deviations. Differences were statistically analyzed by Student's *t*-test. n.s., no statistical significance.

To examine renal function and oxidative stress in elderly and young ICR mice and also in *miR-29b*- and siControl-administered ICR mice, we measured the levels of creatinine, hexanoyl-lysine adduct (HEL) and 8-hydroxy-2′-deoxyguanosine (8OH-dG) in plasma: creatinine is an index of renal function, and HEL and 8OH-dG are stress markers. An increasing tendency of either HEL or 8OH-dG in elderly mice compared with young mice was observed (Fig. 7); this may suggest an increase in oxidative stress with increasing age. As for the others, there was no significant difference observed.

## Discussion

Gene regulation involving miRNAs plays an essential role in various life functions and activities. Senescence is a general deterioration in functioning, in which many biological changes including the alteration of gene expression are presumed to be involved. In this study we examined miRNAs in normal elderly ICR mice as well as in *klotho*(−/−) mice, a senescence-model animal, and the findings revealed that the *miR-29* members were upregulated in both elderly and senescence-model mice. Interestingly, the upregulation of miRNAs was seen in major tissues of elderly ICR as well as *klotho*(−/−) mice except for the *klotho*(−/−) brain [10]; thus, *miR-29* upregulation may be a common event associated with aging. As for why the *klotho*(−/−) brain differed from other tissues, this issue remains to be resolved.

In this study, we specifically used outbred ICR mice as a normal mouse strain with natural aging. Since ICR mice possess heterogeneous genomes, ICR may be a suitable lab animal for studying natural aging like the aging of human beings, who also have heterogeneous genetic backgrounds. While there are differences in genetic background between mouse and human, basic genes or gene regulatory systems essential for the fundamental mechanism of natural aging, if any, may be common between them, and such genes or regulatory systems may be capable of becoming useful molecular indicators for studying natural aging. In the current study using outbred ICR mice, we found that the *miR-29* members were upregulated in elderly ICR mice although there were more or less individual differences in the degree of their upregulation. The *miR-29* upregulated in elderly individuals may be closely related to natural aging, and this raises the possibility that upregulation of *miR-29* may be encoded as a genetic program associated with aging.

As with previous studies, our current study indicate that the *type IV collagen (Col4α1–α6)* genes are potential targets of *miR-29*. Type IV collagen is localized in the basement membrane and involved in the maintenance of the structure of extracellular matrix. Our current study further indicates that the *type IV collagen* transcript and protein levels in elderly mouse tissues are less than in young tissues; this may represent weakening of the basement membranes in elderly tissues, and may be compatible with the progression of aging. While, the transcription attenuation of *Col4α1–α6* may be a major cause of the reduction of type IV collagen in elderly tissues, our findings suggest another contribution of *miR-29* to the suppression of type IV collagen. Thus, *miR-29* appears to be involved in the reduction of *type IV collagen* transcripts and other gene transcripts carrying putative binding sites for *miR-29*. Taken together, the *miR-29* upregulated in elderly tissues may work as a mediator in RNA degradation of target transcripts as well as in translation inhibition, which is the primary function of miRNAs.

**Figure 8:**
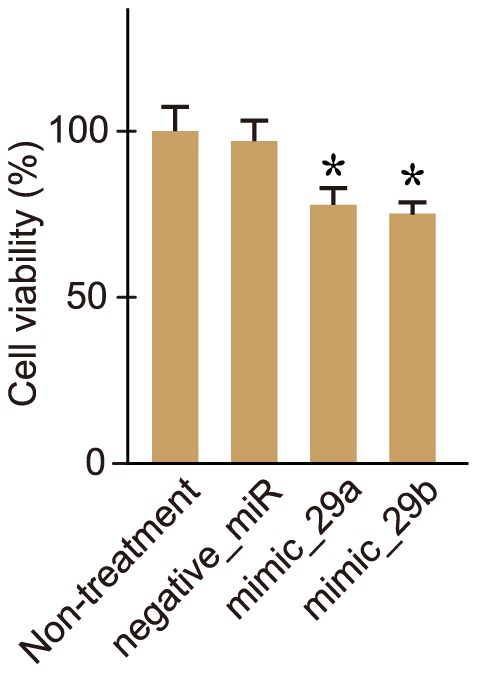
Cell viability of N2a cells treated with *miR-29* mimics. The *miR-29* mimics (mimic_29a and mimic_29b) and a negative control miRNA (negative_miR) were transfected into N2a cells as in Fig. 6A. Three days after transfection, cell viability assay was performed. Data are averages of four measurements. Error bars represent standard deviations. Difference between non-treated cells and each of the treated cells was statistically analyzed by ANOVA, followed by the Dunnett's test (* *P*<0.05).

Previous *in vitro* studies using cultured mammalian cells indicated that *miR-29* is associated with cellular senescence: the *miR-29* members were upregulated during cellular senescence in cultured mammalian cells [21], and appeared to be directly and indirectly engaged in gene regulation of cellular senescence-related *B-Myb* and *p53* genes, respectively [22]–[25]. In the current study, the *miR-29*-administered N2a and HEK293 cells appeared to be weakened (Fig. 8 and Fig. S4); this might reflect cellular senescence triggered by an increase in the amount of *miR-29* in the cells. Taken together, multiple senescence-associated genes may be regulated by the *miR-29* members, and a gene regulatory network may participate in the progression or signs of aging.

Finally, *miR-29* may be a common miRNA coupled to the progression of aging in various tissues, and may have a key role in senescence. Further studies on the relationship between *miR-29* and the aging process need to be carried out. Additionally, confirmatory experiments to see whether human beings deliver similar results need to be performed. If the results are positive, the *miR-29* members and also their target *Col4α1–α6* genes could become useful molecular markers of general senescence.

## Supporting Information

Figure S1 **Reproducibility of miRNA expression profiles.** Expression profile analyses of miRNAs in indicated tissues of *klotho*-deficient [*klotho(−/−)*] and wild-type littermate mice were carried out using the *Genopal*®-MICM DNA chip, and duplicated with two different individual mice (No. 1 and No. 2). The expression profile data were compared to each other by scatter-plot graphs. The expression data of miRNAs were represented by hybridization signal intensities and indicated by arbitrary units.
(TIF)

Figure S2 **Evaluation of** ***in vivo*** **knockdown potency mediated by endogenous** ***miR-29*** **.** The reporter plasmids (Fig. 2A) and *β-galactosidase* expression plasmid as a control were systemically administered to young (5 week-old; 5w) ICR mice and examined as described in Fig. 3. The normalized levels of luciferase activities in liver and kidney were plotted by scatter graphs. As a result, the data obtained with psiCheck2 (empty vector) as a control appeared to be poorly-reproducible in the liver; in contrast, the kidney data remained stable. Therefore, the evaluation of *in vivo* knockdown potency mediated by endogenous *miR-29* in the liver could not be carried out because the control data varied widely. Averaged values of the data are indicated by bars (−).
(TIF)

Figure S3 **Gene expression profiles in** ***miR-29*** **-treated HEK293 cells.** MISSION microRNA Mimics (mimic_29a and mimic_29b) and a negative control miRNA (negative_miR) were transfected into HEK293 cells as in Fig. 6A. Twenty four hours after transfection, total RNAs were extracted and subjected to RT-qPCR followed by analysis using the delta-delta Ct method with the expression level of *GAPDH* as a control. The data were further normalized to the data obtained with the negative_miR. Data are average of four independent measurements. Error bars represent standard deviations. The genes examined are indicated: *COL4A1*, *COL4A2*, *DICER1*, *LAMC1*, *CABIN1* and *DNMT1*. Differences between the negative control and mimic_29a or _29b were statistically analyzed by ANOVA, followed by Dunnett's test (* *P*<0.05). n.s., no statistical significance.
(TIF)

Figure S4 **Cell viability of HEK293 cells treated with miR-29 mimics.** The *miR-29* mimics (mimic_29a and mimic_29b) and a negative control miRNA (negative_miR) were transfected into HEK293 cells as in Figure S3. Three days after transfection, cell viability was examined as in Fig. 8. Data are averages of four measurements. Error bars represent standard deviations. Difference between non-treated cells and each treated cells was statistically analyzed by ANOVA, followed by Dunnett's test (* *P*<0.05).
(TIF)

Table S1 **Difference in the expression of miRNAs between** ***klotho*** **-deficient and wild-type mice.** Hybridization signal intensities obtained from DNA chip analysis [duplicated experiments (Exp.) 1 and 2] are indicated. Fold changes (FC) in the expression of miRNAs between the *klotho* (Kl) and wild-type (Wt) mice are calculated.
(TIF)
