# Supplementary figures and images for: Reduction of Type IV Collagen by Upregulated miR-29 in Normal Elderly Mouse and klotho-Deficient, Senescence-Model Mouse
Source: PLoS One. 2012 Nov 6;7(11):e48974. doi: 10.1371/journal.pone.0048974 (PMC3490916; doi:10.1371/journal.pone.0048974)

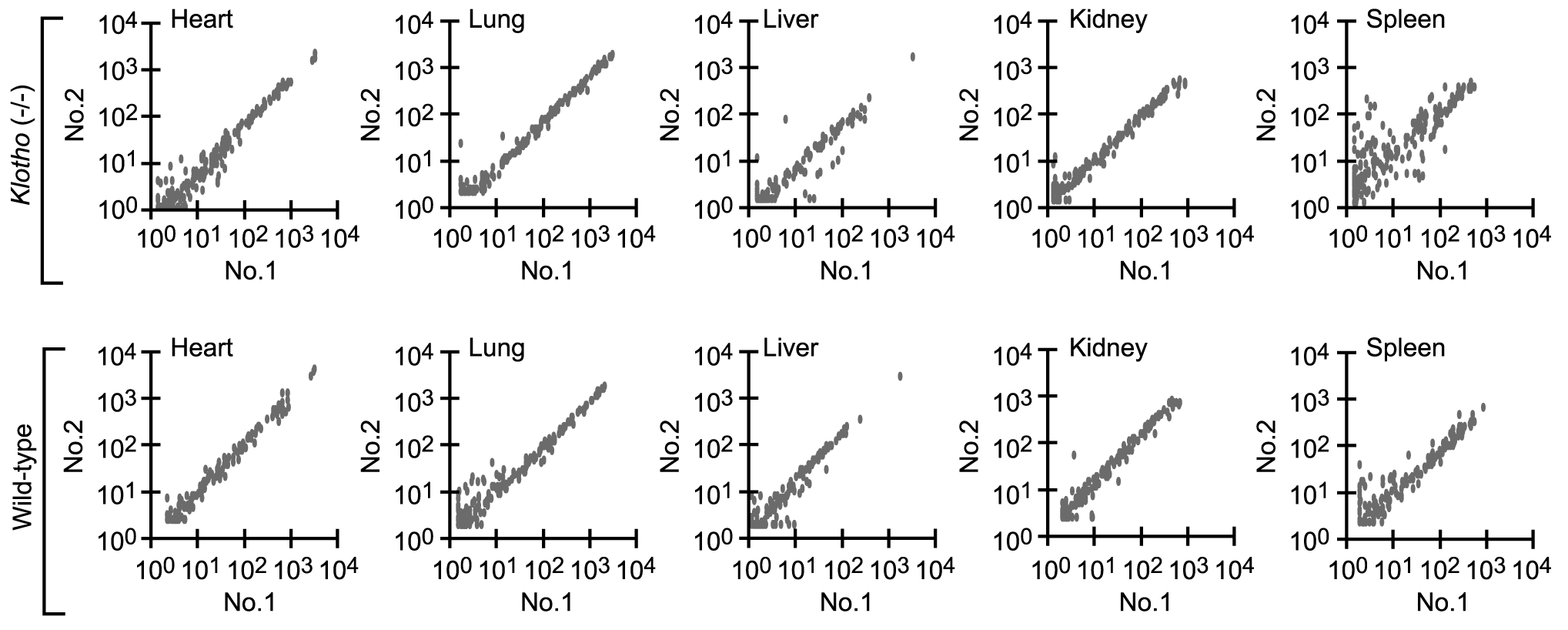

Supplement: Figure S1 — Reproducibility of miRNA expression profiles. Expression profile analyses of miRNAs in indicated tissues of klotho-deficient [klotho(−/−)] and wild-type littermate mice were carried out using the Genopal®-MICM DNA chip, and duplicated with two different individual mice (No. 1 and No. 2). The expression profile data were compared to each other by scatter-plot graphs. The expression data of miRNAs were represented by hybridization signal intensities and indicated by arbitrary units. (TIF) [file pone.0048974.s001.tif]

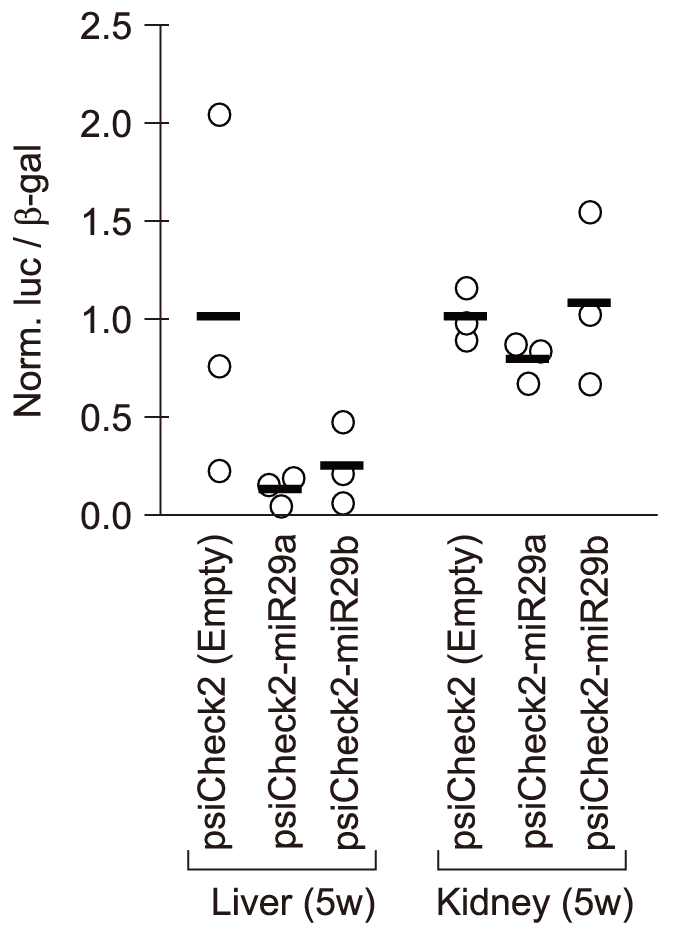

Supplement: Figure S2 — Evaluation of in vivo knockdown potency mediated by endogenous miR-29 . The reporter plasmids (Fig. 2A) and β-galactosidase expression plasmid as a control were systemically administered to young (5 week-old; 5w) ICR mice and examined as described in Fig. 3. The normalized levels of luciferase activities in liver and kidney were plotted by scatter graphs. As a result, the data obtained with psiCheck2 (empty vector) as a control appeared to be poorly-reproducible in the liver; in contrast, the kidney data remained stable. Therefore, the evaluation of in vivo knockdown potency mediated by endogenous miR-29 in the liver could not be carried out because the control data varied widely. Averaged values of the data are indicated by bars (−). (TIF) [file pone.0048974.s002.tif]

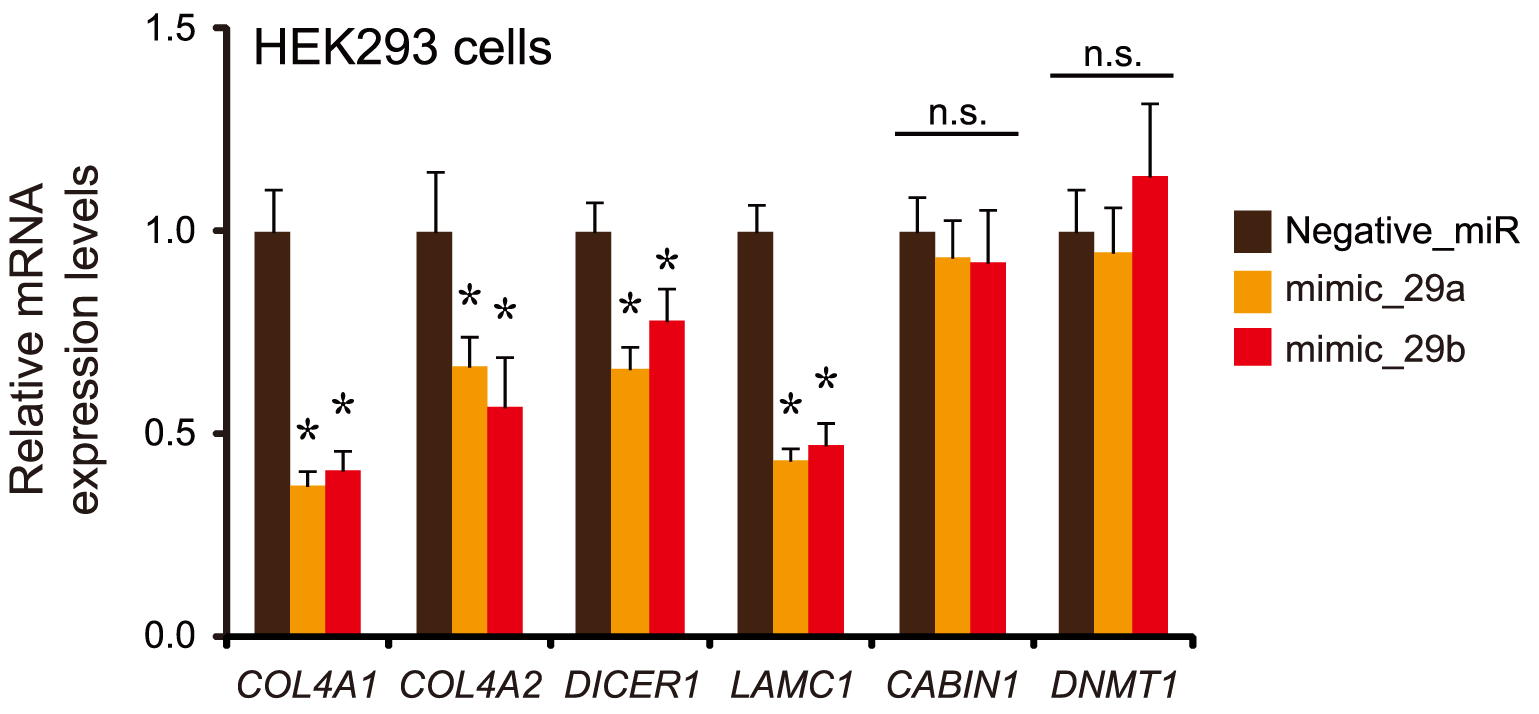

Supplement: Figure S3 — Gene expression profiles in miR-29 -treated HEK293 cells. MISSION microRNA Mimics (mimic_29a and mimic_29b) and a negative control miRNA (negative_miR) were transfected into HEK293 cells as in Fig. 6A. Twenty four hours after transfection, total RNAs were extracted and subjected to RT-qPCR followed by analysis using the delta-delta Ct method with the expression level of GAPDH as a control. The data were further normalized to the data obtained with the negative_miR. Data are average of four independent measurements. Error bars represent standard deviations. The genes examined are indicated: COL4A1, COL4A2, DICER1, LAMC1, CABIN1 and DNMT1. Differences between the negative control and mimic_29a or _29b were statistically analyzed by ANOVA, followed by Dunnett's test (* P<0.05). n.s., no statistical significance. (TIF) [file pone.0048974.s003.tif]

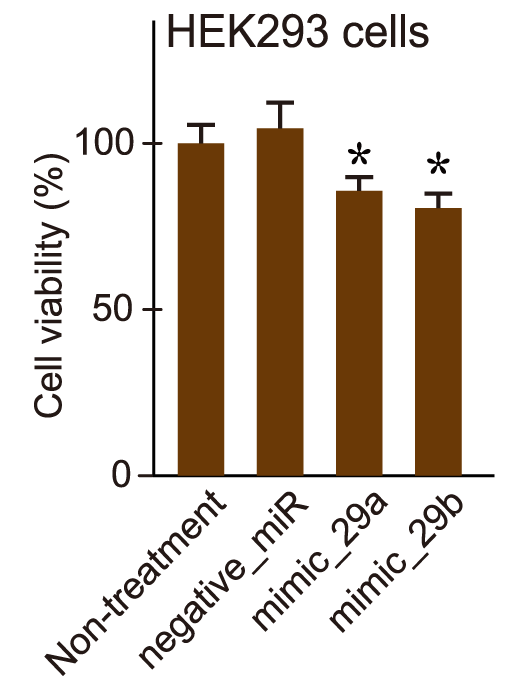

Supplement: Figure S4 — Cell viability of HEK293 cells treated with miR-29 mimics. The miR-29 mimics (mimic_29a and mimic_29b) and a negative control miRNA (negative_miR) were transfected into HEK293 cells as in Figure S3. Three days after transfection, cell viability was examined as in Fig. 8. Data are averages of four measurements. Error bars represent standard deviations. Difference between non-treated cells and each treated cells was statistically analyzed by ANOVA, followed by Dunnett's test (* P<0.05). (TIF) [file pone.0048974.s004.tif]

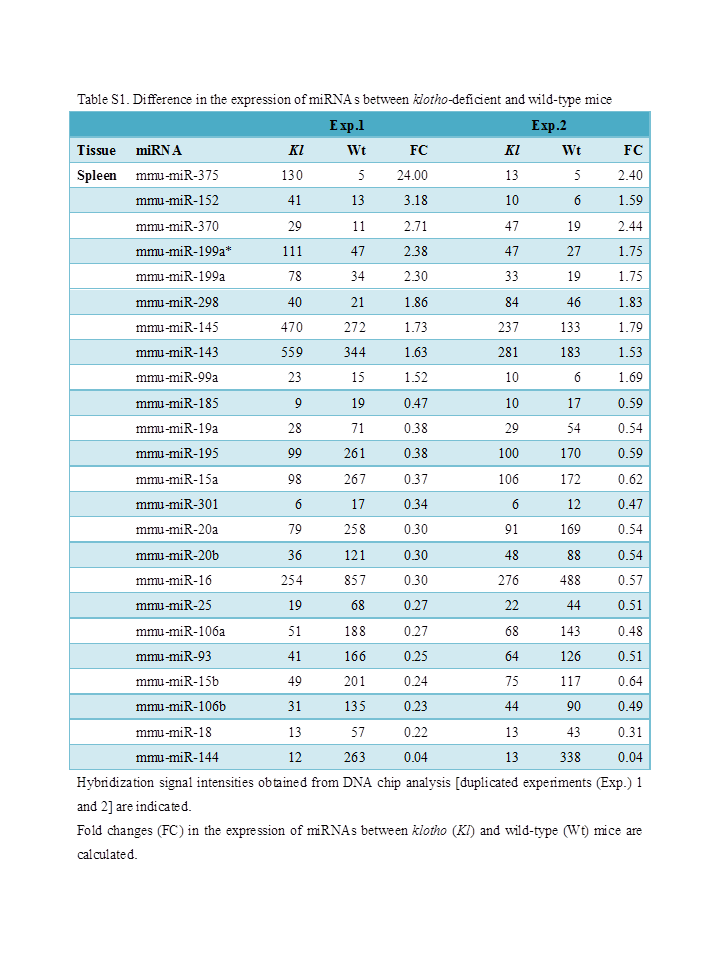

Supplement: Table S1 — Difference in the expression of miRNAs between klotho -deficient and wild-type mice. Hybridization signal intensities obtained from DNA chip analysis [duplicated experiments (Exp.) 1 and 2] are indicated. Fold changes (FC) in the expression of miRNAs between the klotho (Kl) and wild-type (Wt) mice are calculated. (TIF) [file pone.0048974.s005.tif]
